# Corosolic acid, a natural triterpenoid, induces ER stress-dependent apoptosis in human castration resistant prostate cancer cells via activation of IRE-1/JNK, PERK/CHOP and TRIB3

**DOI:** 10.1186/s13046-018-0889-x

**Published:** 2018-09-03

**Authors:** Bo Ma, Hang Zhang, Yu Wang, Ang Zhao, Zhiming Zhu, Xiaowen Bao, Yang Sun, Lin Li, Qi Zhang

**Affiliations:** 1 School of Pharmaceutical Sciences, Nanjing Tech University, Nanjing, 210009 People’s Republic of China; 2 Institute of Advanced Materials (IAM), Nanjing Tech University, Nanjing, 210009 People’s Republic of China

**Keywords:** Corosolic acid (CA), Endoplasmic reticulum stress (ER stress), CCAAT-enhancer-binding protein homologous protein (CHOP), Tribbles homolog 3 (TRIB3), Castration resistant PCa (CRPC), Protein kinase RNA-like endoplasmic reticulum kinase (PERK)

## Abstract

**Background:**

The development of potent non-toxic chemotherapeutic drugs against castration resistant prostate cancer (CRPC) remains a major challenge. Corosolic acid (CA), a natural triterpenoid, has anti-cancer activity with limited side effects. However, CA anti-prostate cancer activities and mechanisms, particularly in CRPC, are not clearly understood. In this study, we investigated CA anti-tumor ability against human CRPC and its mechanism of action.

**Methods:**

The cell apoptosis and proliferation effects were evaluated via MTT detection, colony formation assay and flow cytometry. Western blot, gene transfection and immunofluorescence assay were applied to investigate related protein expression of Endoplasmic reticulum stress. A xenograft tumor model was established to investigate the inhibitory effect of CA on castration resistant prostate cancer in vivo.

**Results:**

The results showed that CA inhibited cell growth and induced apoptosis in human prostate cancer cell (PCa) line PC-3 and DU145, as well as retarded tumor growth in a xenograft model, exerting a limited toxicity to normal cells and tissues. Importantly, CA activated endoplasmic reticulum (ER) stress-associated two pro-apoptotic signaling pathways, as evidenced by increased protein levels of typical ER stress markers including IRE-1/ASK1/JNK and PERK/eIF2α/ATF4/CHOP. IRE-1, PERK or CHOP knockdown partially attenuated CA cytotoxicity against PCa cells. Meanwhile, CHOP induced expression increased Tribbles 3 (TRIB3) level, which lead to AKT inactivation and PCa cell death. CHOP silencing resulted in PCa cells sensitive to CA-induced apoptosis.

**Conclusion:**

Our data demonstrated, for the first time, that CA might represent a novel drug candidate for the development of an anti-CRPC therapy.

**Electronic supplementary material:**

The online version of this article (10.1186/s13046-018-0889-x) contains supplementary material, which is available to authorized users.

## Background

Prostate cancer (PCa) is the second leading cause of cancer-related death among men in the western world. At present, radiation, surgery or pharmacological androgen deprivation therapy is the most common treatments against PCa, especially for hormone-dependent PCa (HDPC). Although many PCa patients have HDPC, unfortunately, the vast majority of them finally become hormonal refractory PCa (HRPC) or castration-resistant PCa (CRPC) patients after 18 to 24 months [1, 2]. CRPC has become one of the difficult problems for urologists and oncologists due to its high metastatic potential, resistance to chemotherapeutic agents and easy relapse. It remains one of the main causes of high mortality in PCa patients. At present, few agents are currently available against CRPC [3, 4]. Therefore, there is a desperate need to discover efficient drugs to treat PCa, in particular those which are effective against CRPC.

The endoplasmic reticulum (ER), as a subcellular organelle, is responsible for Ca^2+^ homeostasis, protein folding and lipid biosynthesis, etc. ER stress response pathways can be induced when accumulation of unfolded proteins and protein misfolding in the ER lumen are caused by oxidative stress, Ca^2+^ disequilibrium, viral infection, diabetes and drugs. It should be noted that ER stress plays significant roles on various biological processes, such as proliferation, metabolism, inflammation, autophagy, and apoptosis [5]. Transient ER stress is protective for cell survival by enhancing of protein degradation, clearance of unfolded proteins and antioxidation. However, severe and sustained ER stress may lead to extrinsic or intrinsic apoptotic pathway. Three protein sensors, such as inositol-requiring enzyme 1(IRE-1), Protein kinase RNA-like endoplasmic reticulum kinase (PERK) and activating transcription factor 6 (ATF6) in ER transmembrane are removed from the chaperone protein glucose-regulated protein78, also known as binding immunoglobulin protein (Bip) when ER stress response pathways is activated [6, 7]. Most recent studies indicate that ER stress plays an important role in apoptosis by two main ways. (1) Sustained IRE-1 activation serves as a positive regulatory factor to phosphorylate apoptosis signal–regulating kinase 1(ASK1) and its downstream target c-Jun NH2-terminal kinase (JNK). This process directly triggers to mitochondrial apoptotic pathway induced-cell death by leading to Bcl-2 and Bcl-xL phosphorylation and their subsequent inactivation [8]. (2) PERK hyperactivation phosphorylates eIF2a, activates ATF4, leading to apoptotic death by finally activating CHOP (CCAAT-enhancer-binding protein homologous protein) [9]. Therefore, agents that induce excessive ER stress have promising antitumor effects.

It is increasingly recognized that activation of the ER stress can be an effective strategy to eliminate cancer cells [10, 11]. The function of prostate, as an important secretory gland, mainly depends on ER and is vulnerable to agents or conditions that cause ER stress [12]. Glucose-regulated protein78-targeting subtilase cytotoxin catalytic subunit results in a high sensitivity of PCa to chemotherapeutic drugs and outstanding anti-cancer activity by increasing CHOP level in Bax-deficient and apoptosis-resistant DU145 PCa cells [13]. Overexpression of Bcl-2 family members is found in CRPC with chemotherapeutic resistance. The combination treatment of Pim kinase inhibitors and antagonists of Bcl-2 family members sensitizes PCa cells to apoptosis by inducing ER stress to result in eIF-2α phosphorylation and increasing expression of CHOP in PCa cells [14]. Thus, the pro-apoptotic ER stress modulation is a potential therapeutic strategy for CRPC treatment [15].

**Fig. 1:**
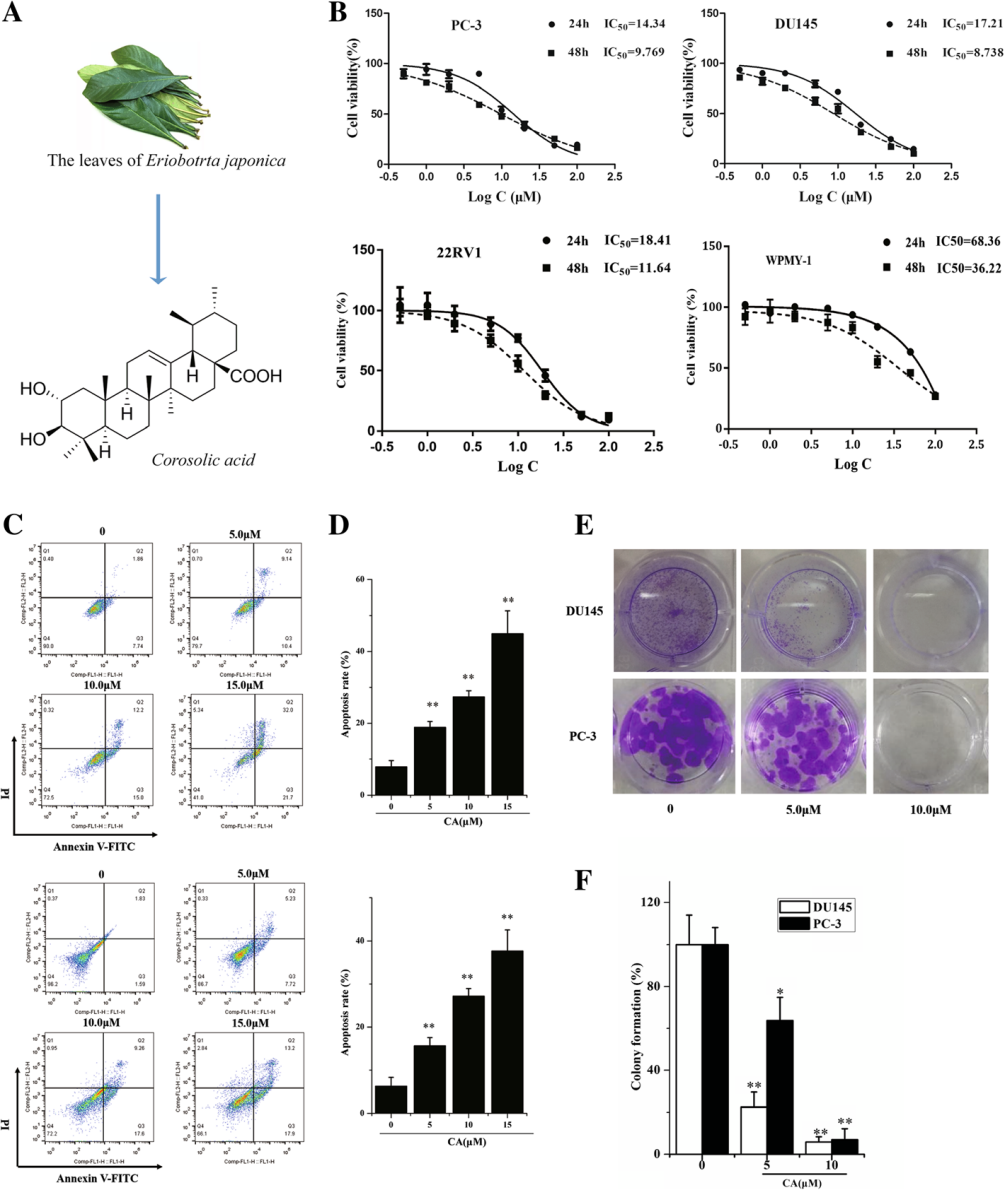
CA suppresses cell proliferation and induces apoptosis in human prostate cancer cells. **a** Structure of the CA molecule. **b** PC-3, DU145, 22rv1 and WPMY-1 cells were treated with various concentrations of CA for 24 and 48 h, cell viability was assayed by MTT assay. **c** Apoptosis index in PC-3 and DU145 cells with 0, 5, 10 and 15 μM CA treatments for 24 h detected by annexinV/PI flow cytometry assay. **d** Statistical analysis result of flow cytometric analysis of apoptosis (both of early and later apoptosis). **e** Cell proliferation was measured by colony formation in 12-well plates with crystal violet staining. Representative photographs are shown. **f** The percentage of colony formation was calculated by defining the number of colonies in the absence of CA as 100%. The results are presented as mean ± SD and described as column chart *p < 0.05 and **p < 0.01 as compared with untreated control

Corosolic acid (CA, Fig. 1a), a pentacyclic triterpene compound, is one of the lipophilic constituents extracted from the leaves of *Eriobotrta japonica* [16], the fruit of *Cratoegus pinnatifida var. psilosa* [17] and the root of *Actinidia chinensis* [18] and has shown excellent effects against diabetes on animal experiments and clinical trials [19]. A research showed that it could enhance glucose uptake in L6 myotubes and facilitates glucose transporter isoform 4 translocation in CHO/hIR cells via up-regulating insulin receptor phosphorylation [20]. CA as the prevention and treatment of obesity and type 2 diabetes agent, has entered III clinical pharmacodynamic evaluation in the Food and Drug Administration (FDA, USA). In recent years, its anti-tumor activity induced more and more concerns [21, 22]. It has shown cytotoxic effects in several tumors including cervical cancer [23], hepatocellular carcinoma [18], glioblastoma [21] and lung adenocarcinoma [22], both in vitro and in some tumor xenograft models in vivo. Increasing number of studies showed that CA modulates many cellular signaling events, including Stat-3 [24], nuclear factor-kappa B [25] and Wnt/β-catenin [26]. However, the roles of CA in PCa remain largely unknown, particularly in CRPC. Currently, it is unclear whether ER stress-mediated apoptotic pathways play a pivotal role in CA-induced cell death.

To date, the anti-tumor molecular CA mechanisms underlying its apoptotic effect in human PCa cells has not yet been determined. In the current study, we firstly explored the effect of CA in the induction of cell death using human prostate cancer cell line PC-3 and DU145 PCa cell lines, which have hormone-independent characteristics. In addition, the underlying mechanisms were also elucidated by investigating the involvement of possible ER stress-dependent apoptosis signaling in response to CA in human PCa. Overall, we are the first providing a direct evidence that the use of CA might combat CRPC and it could be used as a promising therapeutic agent.

## Methods

### Materials

DAPI 3-(4, 5-dimethylthiazol-2-yl)-2, 5-diphenyl tetrazolium, MTT 3-(4, 5-dimethylthiazol-2-yl)-2, 5-diphenyltetrazolium bromide and Hoechst 33258 were obtained from Sigma (St. Louis, MO, USA). CA (purity: 98%) was purchased from Yuanye Biological Technology Co., Ltd. (Shanghai, China). Purchase, dilution and storage condition of primary antibodies and second antibodies were listed in Additional file 1: Table S1. Hematoxylin, bovine serum albumin (BSA), crystal violet, TritonX-100 and diaminobenzidine (DAB) were obtained from Beyotime (Beyotime, Jiangsu, China)**.** The SP600125, SB203580, LY294002, and Z-VAD-FMK were purchased from Selleck Chemicals (Houston, Texas, USA). Matrigel Matrix was purchased from Corning (Corning, NY 14831 USA).

### Cell culture

The human prostate cancer cell line 22RV1, PC-3 and DU145 as well as normal prostate cells (WPMY-1) were obtained from the American Type Culture Collection (ATCC, USA). PC-3 were cultured in F12 K media (Sigma, USA), DU145 was cultured in MEM media (Hyclone, USA), 22RV1 was cultured in 1640 media (Hyclone, USA) and WPMY-1was cultured in DMEM media with high glucose (Hyclone, USA). These media all contains 10% fetal bovine serum (FBS; Hyclone, USA), 10 U/mL penicillins and 100 mg/L streptomycin at 37 °C in a humidified atmosphere of 5% CO_2_. The medium was changed every 2–3 days.

### Measurement of cell viability

The PC-3, DU145, 22RV1 and WPMY-1 cells were seeded in a 96-well plate at a density of 1 × 10^4^ cells/well. Twenty-four hours later, the cells were treated with control (0.1% DMSO) or various concentrations CA. The cell viability was determined using MTT assay, as our previous report [27]. The absorbance at 492 nm was determined in each well by a microplate reader (Thermo Multiskan MK3, Helsinki, Finland).

### Hoechst 33258 staining assay

For Hoechst staining assay, PC-3 and DU145 cells treated with various concentrations of CA (0, 5, 10 and 15 μM) or 0.1% DMSO. After 12 h, the cells were fixed for 20 min using 4% Paraformaldehyde and then were stained with 5 μg/mL Hoechst 33258 dye for 5 min. Stained cells were observed under fluorescence microscope after washing three times with PBS. Cells with bright, fragmented and condensed nuclei were identified as apoptosis cells.

### AnnexinV-FITC/PI double staining assay

The PC-3 and DU145 cells were seeded in 6-well plates at a density of 1.0 × 10^6^ cells/well, and then treated with CA (0, 5, 10 and 15 μM) after inoculation for 24 h. Apoptosis rate was determined via flow cytometry (Becton Dickinson, San Jose, CA, USA) using the Annexin V-FITC Apoptosis Detection kit (KeyGEN Biotech, Jiangsu, China) according to the manufacturer’s instructions. The Annexin V-FITC (−)/PI (−) (lower left quadrant) were defined as normal cells, the Annexin V-FITC (+)/PI(−) cells (lower right quadrant) as early apoptotic cells, Annexin V-FITC (+)/PI(+) (upper right quadrant) as late apoptotic cells, and Annexin V(−)/PI(+) (upper left quadrant) as necrotic cells.

### Colony formation assay

The PC-3 and DU145 cells were seeded in a 12-well plate at a density of 1000 cells/well. They were cultured overnight and then treated with CA at various concentrations (5 and 10 μM) for 14 days. The cell monolayer was washed twice with PBS, fixed with 4% paraformaldehyde and stained with 1% crystal violet solution to count the number of colonies. The colonies were considered as survivors and counted only when they contained more than 50 cells. The number of colonies in each well was counted under an inverted microscope.

### Measurement of mitochondrial membrane potential (ΔΨm)

Mitochondrial membrane potential (ΔΨm) was determined by the JC-1 probe method according to the manufacturer’s protocol (Beyotime, Jiangsu, China). In brief, after the treatment of various concentrations CA (0, 5, 10 and 15 μM), the cells were digested, harvested, washed and stained with 10 μg/mL JC-1 at 37 °C for 30 min in the dark. Subsequently, stained cells were washed, resuspended, and subjected to flow cytometry analysis (Becton Dickinson, San Jose, CA, USA).

### Transfection of small interfering RNA (siRNA)

The PC-3 and DU145 cells were seeded onto a 6-well plate at a density of 5× 10^5^ cells/well. The siRNA was synthesized from GenePharma Company (Shanghai, China). The PC-3 and DU145 cells were transiently transfected with PERK siRNA (sense: 5′-GUGGAAAGGUGAGGUAUAUTT′, antisense: 5′-AUAUACCUCACCUUUCCACTT-3′);

CHOP siRNA (sense: 5′GCGCAUGAAGGAGAAAGAATT′, antisense: 5′-UUCUUUCUCCUUCAUGCGCTT-3′); IER-1 siRNA (5′- CAACCUCUCUUCUGUAUCUTT -3′, antisense: 5′- AGAUACAGAAGAGAGGUUGTT-3′) [28]; or scramble siRNA (sense: 5’-UUCUCCGAACGUGUCACGUTT-3′; antisense:5’-ACGUGACACGUUCGGAGAATT-3′) as negative control, respectively, using Lipofectamine 2000 (Applied Biological Materilas Inc., Canada), according to the manufacturer’s instructions. After siRNA transfection overnight, cells were either collected for validation by Western blot analysis or for subsequent experiments.

### Real-time PCR

The PC-3 and DU145 cells were seeded onto a 60 mm culture dish at a density of 1 × 10^6^ cells. They were cultured to adhere overnight and then treated with CA. Total RNA from cultured cells was extracted using Trizol (TaKaRa Biotechnology. Dalian). The cDNA was synthesized with a cDNA synthesis kit according to the manufacturer’s protocols (Applied Biological Materilas Inc., Canada). Quantitative real-time PCR was performed by BrightGreen 2X qPCR Master Mix kit (Applied Biological Materilas Inc., Canada) and CFX96 Touch Real-Time PCR detection system (Bio-rad, USA) to conform the mRNA expression. The primer sequences for CHOP, TRIB3 and GAPDH were listed in Additional file 2: Table S2. The comparative cycle of threshold fluorescence (Ct) method was adopted as well as the relative transcript amount of the target gene was normalized to that of GAPDH and calculated using the 2^-ΔΔCt^ method.

### Protein extraction and western blot analysis

After treatment, total proteins of PC-3 and DU145 cells were lysed in RIPA lysis buffer (Beyotime, Jiangsu, China) on ice according to the manufacturer’s protocol. Protein concentrations were detected by BCA protein assay kit (Bio-Rad, Hercules, CA, USA). Equal mounts of protein extract (40 μg) extracted from cells were applied to 12, 10 or 8% SDS-polyacrylamide gels. The PVDF membranes were blocked with TBST containing 5% bovine serum albumin (BSA) for 2 h at room temperature. And then the membranes were incubated with primary antibody overnight at 4 °C (Dilution ratio of primary antibody was listed in Additional file 1: Table S1). And then the membranes were washed thrice by TBST and incubated with diluted the enzyme horseradish peroxidase (HRP)-conjugated secondary antibodies (1:6000) for 1.5 h at room temperature. After extensive washing, the signals were detected by ECL reagent (Millipore, Billerica, MA, USA) and quantified using the Clinx Chemi Image Analysis software (Clinx Science, Shanghai, China). GAPDH was used as loading control.

### Immunofluorescent assay

To investigate the nuclear translocation and expression of CHOP in human prostate cancer after CA treatment, immunofluorescent assay was performed in current research. The PC-3 and DU145 cells were seeded onto small glass dishes at a density of 1 × 10^5^ cells/well. After CA treatment for 12 h, the cells were fixed with 4% paraformaldehyde for 30 min, permeabilized with 0.5% TritonX-100 for 10 min, and blocked with TBST containing 5% BSA for 1 h at 37 °C. The cells were incubated with CHOP (1:200) overnight at 4 °C. Then, the cells incubated for 1 h at 37 °C with DyLight 488-conjugated Affini-Pure goat anti-rabbit IgG secondary antibodies, after they were washed for three times (10 min each). Cell nuclei were stained with DAPI for 5 min in darkroom. Finally, the cells were analyzed by a Zeiss confocal laser microscope (ZEISS LSM 700, Carl Zeiss Jena, Germany).

For co-localization study of cytochrome C and mitochondria, cells were first incubated with MitoRed (100 nM, KeyGEN Biotech, Jiangsu, China), a mitochondrial stain, for 30 min at 37 °C after treatment with CA, and then fixed in paraformaldehyde. The following steps are similar to the above-mentioned method.

### Plasmid transfection

The GV141-PERK and GV141 CHOP vector plasmids were obtained from Genechem Company (Shanghai Genechem Co., LTD. China) Transfections were performed using DNAfectin™ Plus Transfection Reagent (Applied Biological Materilas Inc., Canada), according to the manufacturer’s protocol. Briefly, PC-3 and DU145 cells were seeded in 6-well culture plates; when approximately 50% confluent, cells were transfected with 2 μg plasmid. After transfection overnight, cells were either harvested for validation by Western blot analysis or for subsequent experiments.

### Xenograft tumor model

All mice were housed under specific pathogen-free conditions at 22 ± 2 °C and 55 ± 5% humidity in a barrier facility with 12-h light–dark cycles. Animals had free access to standard mouse chow and water ad libitum. All procedures performed on the animals in this study were approved (NO. NJTECH-AE-2017006) the Guidelines for the Animal Ethics Committee of Nanjing Tech University. After 1 week’s acclimation, 2 × 10^6^ PC-3 cells suspended in 0.1 ml (free F12 K medium: Matrigel = 1:1) were subcutaneously injected into the right flank of each mouse (*n* = 6).

When xenografts reached a volume of 100 mm^3^, the animals were randomized into 3 groups (n = 6) and received the corresponding treatments as the following: Control group (vehicle: 10% DMSO+ 90% Saline) and CA treatment group (10 and 20 mg/kg, respectively) by intraperitoneal injection for every 2 days. Tumor sizes were measured for every 2 days to monitor dynamic changes in tumor growth, and tumor volumes were calculated by a standard formula: length×width^2^/2. After the treatment for 14 days, the animals were sacrificed as well as the tumor tissues were isolated for further study. Other organs (heart, liver, spleen, lung and kidney) were collected for Histopathologic study with staining with hematoxylin and eosin (H & E) to evaluate toxicity of CA, as previously reported methods [27].

### Terminal-deoxynucleoitidyl transferase mediated Nick end labeling (TUNEL) assay

Tumor tissue apoptosis was investigated with the TUNEL assay (Beyotime, Jiangsu, China), according to the manufacturer’s instructions. The stained tissues were visualized under a fluorescence microscope.

### Immunocytochemistry

Tumor tissues were fixed with 4% paraformaldehyde for 30 min, dehydrated with an ethanol series, treated with xylene, mounted in paraffin and sliced. The sections were deparaffinized, rehydrated, and incubated with 3% H_2_O_2_ in methanol. And then they were soaked in sodium citrate buffer (pH 6.0) and were heated by microwave oven to achieve antigen retrieval. Subsequently, these sections were blocked with 10% goat serum for 1 h and were incubated with primary antibody at 4 °C overnight. After being washed three times with TBST, the slides were sequentially incubated with Horseradish peroxidase–polymer anti-rabbit antibody for 30 min. In the end, the slides were stained for using DAB, and the sections were then counter stained with hematoxylin. Finally, the slides were analyzed with an inverted microscope (Nikon ECLIPSE 80i, Nikon, Tokyo, Japan).

### Statically analysis

Data were presented as Mean ± standard deviation (SD). Statistical analyses were performed using One-way ANOVA. Differences at *p* < 0.05 were considered statistically significant.

## Results

### CA suppresses cell proliferation and induces apoptosis in human PCa cell line

MTT assay was used to investigate the effect of CA on PCa cell viability. The results suggested that PCa cell viability was efficiently inhibited after CA treatment for 24 and 48 h and the inhibition rates were both concentration- and time-dependent. CA half-maximal inhibitory concentration (IC_50_) on PC-3 and DU145 cells is shown in Fig. 1b. Therefore, all subsequent studies were performed using CA at the concentration of 5, 10, and 15 μM on PC3 and DU145 cells, according to the IC_50_ values. Differently from PCa cells, WYMY-1 cells showed less apoptosis even at 15 μM CA exposure (Fig. 1c). Apoptosis induced by CA was assessed by Hoechst 33258 staining Additional file 4: Figure S1. A large number of cells displayed bright nucleus, nuclear condensation and apoptotic bodies when they were exposed to 15 μM CA for 24 h (Fig. 1d). Annexin V-FITC/PI staining was further used to determine the apoptotic rate induced by CA treatment. CA exposure for 24 h resulted in a concentration-dependent increase in both early and late apoptosis in PC-3 and DU145 cells (Fig. 1f). The colony formation assay was employed to evaluate tumor cell proliferation. Data indicated that CA treatment decreased PCa proliferation rate in vitro (Fig. 1g). These data indicated that CA could exert a pronounced cell growth-inhibitory effect on human PCa cells. In addition, CA at quite lower levels inhibited the migration and invasion of PCa cells (Data of migration and invasion was listed in Additional file 3).

### CA induces apoptosis in human PCa cells by mitochondrial apoptotic pathway

**Fig. 2:**
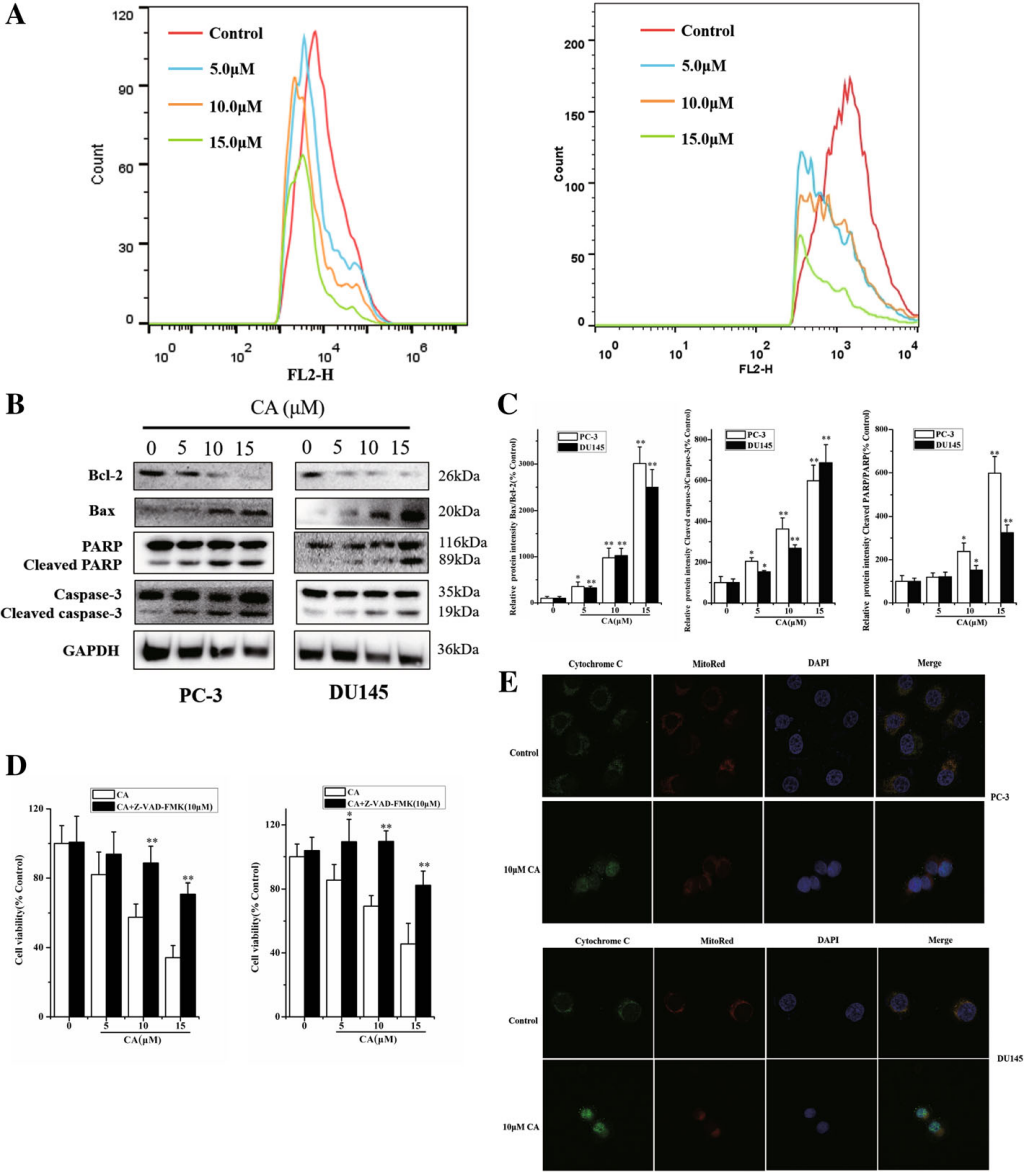
Effect of CA on mitochondria dependent apoptosis in human prostate cancer cells. **a** Mitochondrial membrane potentials (ΔΨm) were assessed with JC-1 staining by flow cytometry. **b** The expression of cell key apoptosis-related proteins was detected by Western-blotting and they were statistically analyzed (**c**). The PC-3 and DU145 cells was pre-treated with caspase inhibitor (10 μM Z-VAD-FMK) for 3 h and followed by incubation with or without CA for 24 h, respectively. **d** The cell viability was determined by MTT assay. The results are presented as mean ± SD and described as column chart *p < 0.05 and **p < 0.01 as compared with corresponding group. **e** Effect of CA induced-Cytochrome c release was analyzed by immunofluorescence staining

To determine whether mitochondrial apoptotic pathway plays a role in CA induced human PCa cell death, the mitochondrial membrane potential was studied using JC-1 staining on PC-3 and DU145 cells. As shown in Fig. 2a, the collapse of the mitochondrial membrane potential was observed in PC-3 and DU145 cells after CA treatment for 24 h in a concentration-dependent manner, as demonstrated by the remarkable accumulation of green fluorescence. Protein expression of related pathways was further analyzed. The Bax (pro-apoptotic protein) and Bcl-2(anti-apoptotic protein) are recognized as important indicator of mitochondrial apoptotic pathway. CA exposure for 24 h resulted in a significant decrease in Bcl-2 levels and increase in Bax levels, confirmed by the Bcl-2/Bax ratio that was significantly decreased (Fig. 2b and c). Cytochrome c is an important mitochondrial inner membrane-associated protein that plays a key role in caspase-induced apoptosis when released into the cytoplasm. Cytochrome c release was examined by immunofluorescence. Untreated human PCa cells displayed a mass of cytochrome c distribution consistent with its mitochondrial location. Exposure to 10uM CA induced a significant cytochrome c release, as shown by a diffuse cytochrome c staining throughout the cytoplasm (Fig. 2e). The modulatory effects of mitochondrial apoptotic pathway including caspase-3 and PARP were also examined in cells treated with CA. Data obtained by western blot showed that CA induced the cleavage of caspase-3 and PARP in a concentration-dependent manner (Fig. 2b and c). Pretreatment of PC3 and DU145 cells with the caspase inhibitor Z-VAD-FMK at 10 μM for 3 h resulted in a complete apoptosis suppression by CA treatment at medium and low concentration compared to control cells, as shown by MTT. Apoptosis induced by high CA concentrations was not completely inhibited by caspase inhibitor, indicating that a non-caspase dependent pathway was partially involved in CA inducing cell death of human PCa cells at higher CA concentration (Fig. 2d). These results indicated that CA induced apoptosis in human PCa cells primarily through mitochondrial apoptotic pathway.

### MAPK and AKT signaling pathways are involved in CA apoptotic effect on human PCa cells

**Fig. 3:**
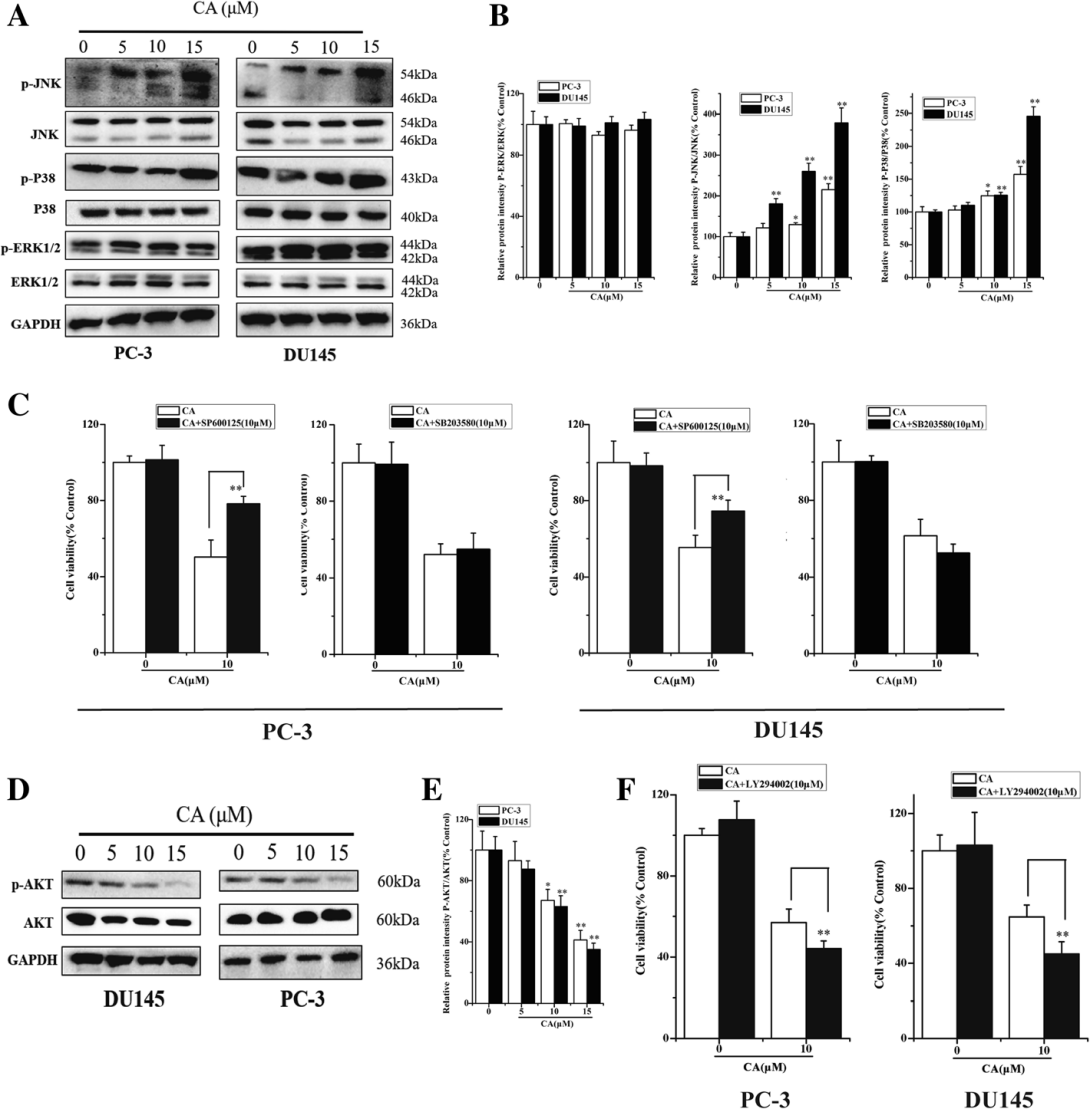
MAPK and AKT signaling pathways are involved in CA apoptotic effect on human PCa cells. MAPKs (**a**) and AKT (**d**) protein expressions were analyzed using western blot. Densitometry scanning analysis for ratio of p-JNK/total JNK, p-P38/total P38 and p-ERK1/2/total ERK1/2 (**b**) as well as p-AKT/total AKT (**e**). Data represent the mean ± SD of three independent experiments. *p < 0.05 and **p < 0.01 as compared with untreated control. **c** Effects of SP600125, SB203580 and LY294002 (**f**) CA-induced apoptosis in cultured cells for 24 h. Cell viability was determined by MTT assay and data represent the mean ± SD of six experiments in each group. *p < 0.05, **p < 0.01 as compared with single CA treated cells

To determine whether MAPK signaling pathway was responsible for CA induced cytotoxicity of PCa cells, we first compared the phosphorylated and total ERK1/2, p38 MAPK and JNK protein levels after CA treatment in PC3 and DU145 cells. Results showed that CA treatment led to a significant phosphorylation increase of both JNK and p38 in a dose-dependent manner, but no change was observed in the total JNK and p38 expression. Neither enhancement nor suppression were found in the total ERK1/2 and phosphorylated ERK1/2 in our experiment (Fig. 3a and b). To elucidate the role of JNK and p38 in CA-induced apoptosis in PCa cells, we pretreated PCa cells with the JNK inhibitor SP600125 or the p38 MAPK inhibitor SB203580 for 6 h before exposing cells to CA. Results showed that SP600125 pretreatment partially suppressed the apoptosis induced by CA. In contrast, p38 MAPK inhibitor SB203580 had no effect on CA induced apoptosis in PCa cells (Fig. 3c). These results indicated the involvement of the JNK pathway in the regulation of apoptosis induced by CA in human PCa cells.

Recent scientific reports indicated that constitutive AKT activation can induce tumor proliferation, progression and aggressiveness. Phosphorylation of AKT was markedly decreased in a dose-dependent manner after CA treatment (Fig. 3d and e). LY294002 pretreatment (a specific PI3K inhibitor that decreases the expression of phosphorylated AKT) in PCa cells induced an increase in the apoptosis of both PC3 and DU145, as evidenced by the reduction in cell viability. These data suggested that JNK and AKT signaling pathways might be involved in the CA apoptotic effect on human PCa cells.

### IRE-1-mediated ASK1-JNK activation plays a key role in CA-induced cell death

**Fig. 4:**
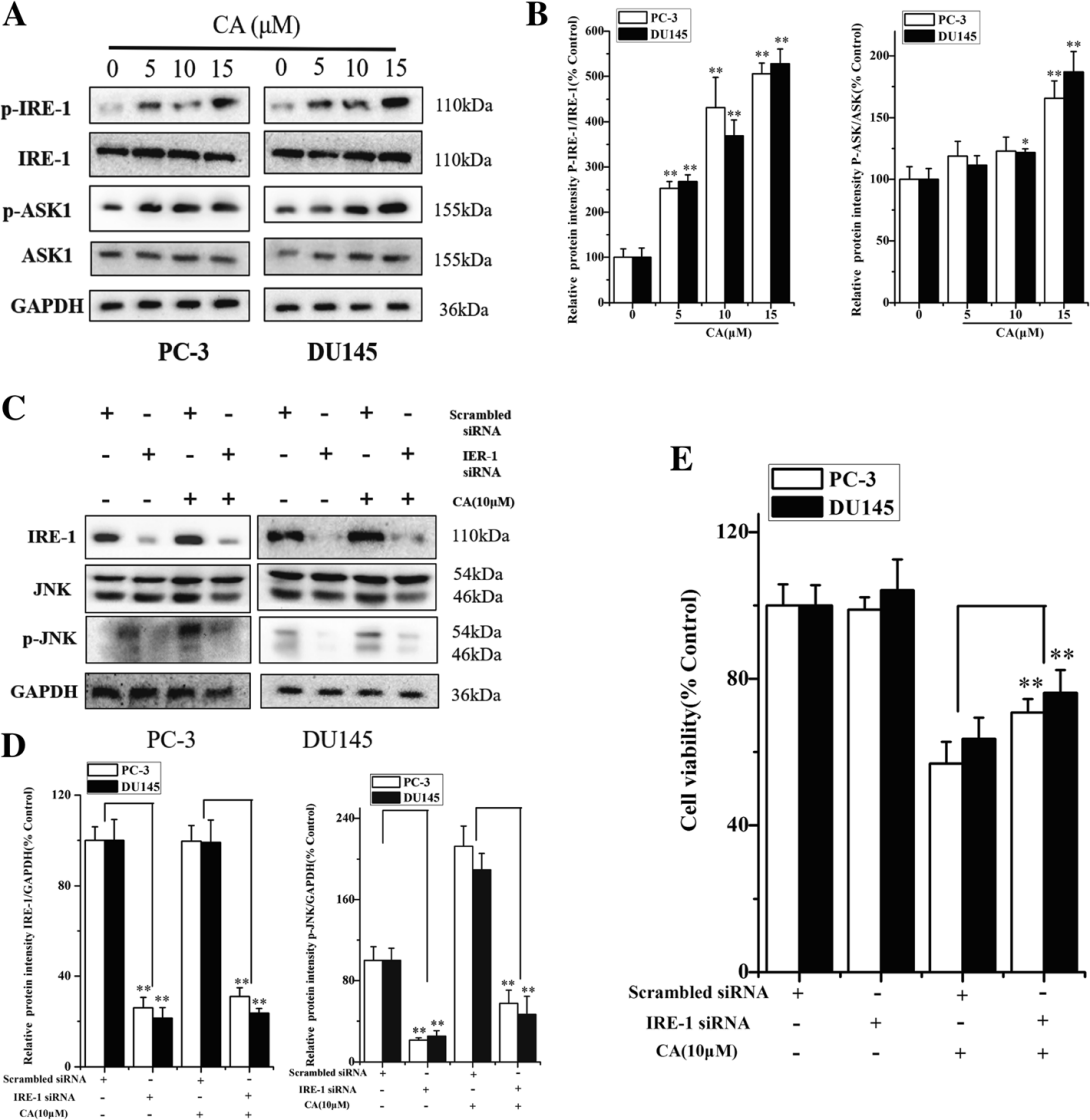
IRE-1-mediated ASK1-JNK activation plays a key role in CA-induced cell death. **a**The PC-3 and DU145 cells were treated, respectively, by CA for 24 h at doses of 5, 10, 15 μM. The p-IRE-1, IRE-1, p-ASK1 and ASK1 protein expression were measured by Western blotting, and the bands were scanned and data of statistically analysis were listed in (**b**). Data represent the mean ± SD of three independent experiments. *p < 0.05 and **p < 0.01 as compared with untreated control. The cells transfected with IRE-1 siRNA or control siRNA were treated with CA (10 μM) for 24 h. The protein expression was measured by western blotting (**c**), Densitometric analysis of protein bands was showed in (**d**). **e**The cell viability was determined by MTT assay. The results are presented as mean ± SD and represent three individual experiments. *p < 0.05 and **p < 0.01 as compared with corresponding group

Activated ER stress induces expression of phospho-JNK, which mediates apoptotic signals. Therefore, to evaluate whether CA induces ER stress-mediated apoptosis in human PCa cells, IRE-1-ASK1-JNK signaling branch of ER stress was investigated in the present research. Results in Fig. 4a and b suggested that CA treatment significantly increased the phosphorylation of both IRE-1 and ASK1 in both cell types. However, no significant change in total IRE-1 and ASK1 expression was observed in PC3 and DU145 cells. IRE-1 recruits ASK1 and leads to JNK activation, which is implicated in ER stress-induced cell death. To validate whether IRE-1 was activated after CA treatment and further induced phospho-JNK (a downstream target of IRE-1), we assessed the effect of IRE-1 silencing on CA-mediated activation of JNK. As shown in Fig. 4c, d and e, IRE-1 knockdown resulted in a significant decrease in JNK activation and inhibition in the decline of cell viability after CA treatment. Taken together, these results suggested that CA induced apoptosis was partly mediated by one of the signaling pathways in ER stress, such as IRE-1-mediated ASK1-JNK activation.

### CA activates the PERK-eIF2a-ATF4 pathway leading to the pro-apoptotic CHOP up-regulation in human PCa cells

**Fig. 5:**
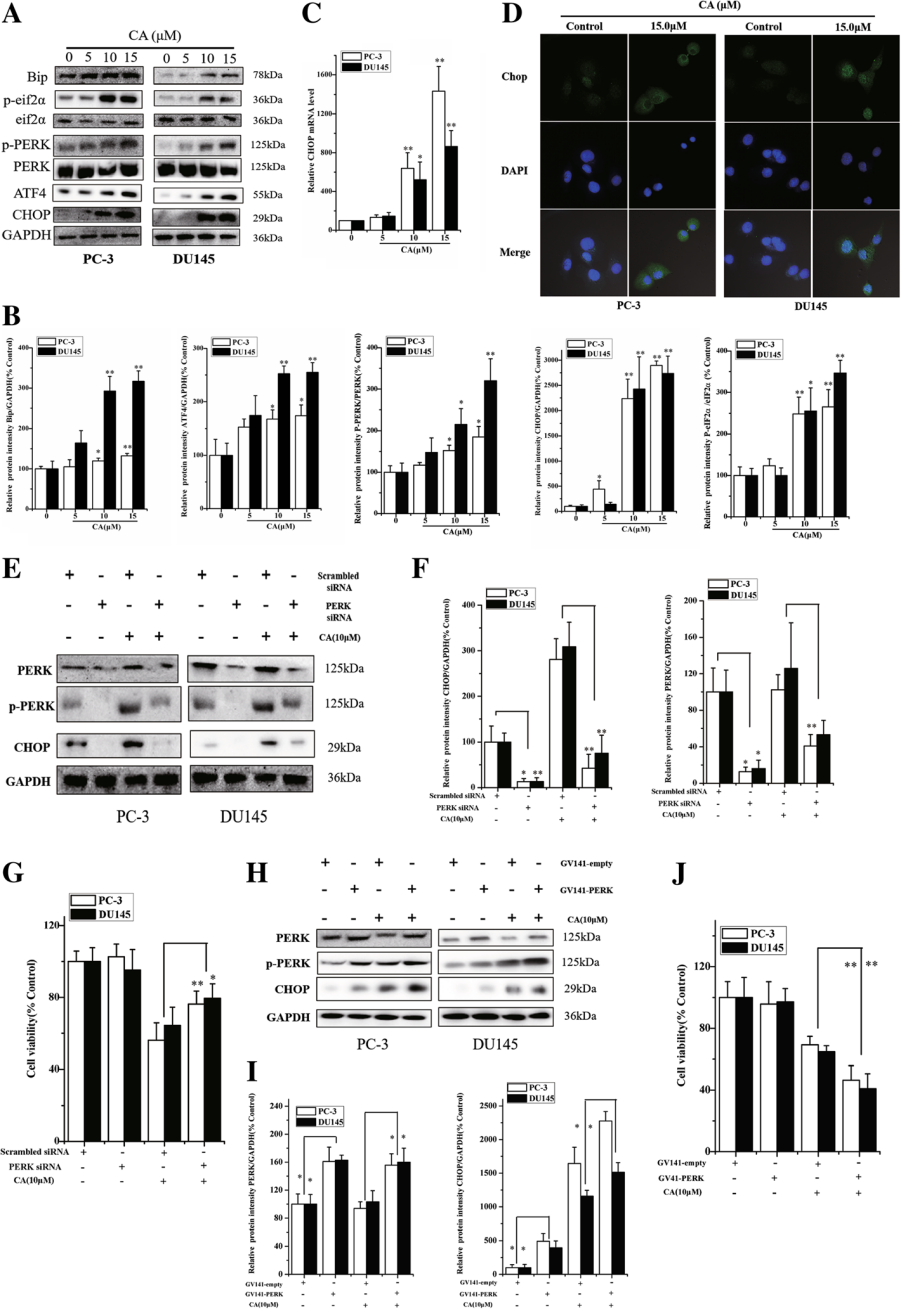
CA activates the PERK-eIF2a-ATF4 pathway leading to the pro-apoptotic CHOP up-regulation in human PCa cells. The PC-3 and DU145 cells were treated with CA at the indicated concentrations (5, 10, 15 μM.) for 24 h. **a** the phosphorylation of PERK, eIF2α and expression levels of Bip, PERK, eIF2α, ATF4 and CHOP were measured by western blot using corresponding antibodies. **b** And Protein levels were quantified by grayscale scan and the GAPDH was used as the loading control. **c** Total RNAs were extracted and subjected to real-time PCR analysis using specific primer sets for CHOP and GAPDH, and the data were normalized to GAPDH expression. The results are presented as mean ± SD and represent three individual experiments. *p < 0.05 and **p < 0.01 as compared with untreated control. **d** Effects of CA induced-CHOP activation analyzed by immunofluorescence staining. The PC-3 and DU145 cells were transfected with PERK siRNA or control siRNA, respectively and treated with CA for 24 h. **e** The protein expression of PERK and CHOP was measured by western blotting. **f** Protein levels were quantified by grayscale scan and the GAPDH was used as the loading control. **g** The cell viability was determined by MTT assay. The results are presented as mean ± SD and represent three individual experiments. *p < 0.05 and **p < 0.01 as compared with corresponding group. **h** Overexpression of PERK in PC-3 and DU145 cells that were treated with CA for 24 h. PERK, p-PERK and CHOP protein expressions were determined by western blot. **i** Protein levels were quantified by grayscale scan and the GAPDH was used as the loading control. **j** MTT assay demonstrated that PERK overexpression aggravated CA-induced migration inhibition of cell viability in PC-3 and DU145. The results are presented as mean ± SD and represent three individual experiments. *p < 0.05 and **p < 0.01 as compared with corresponding group

**Fig. 6:**
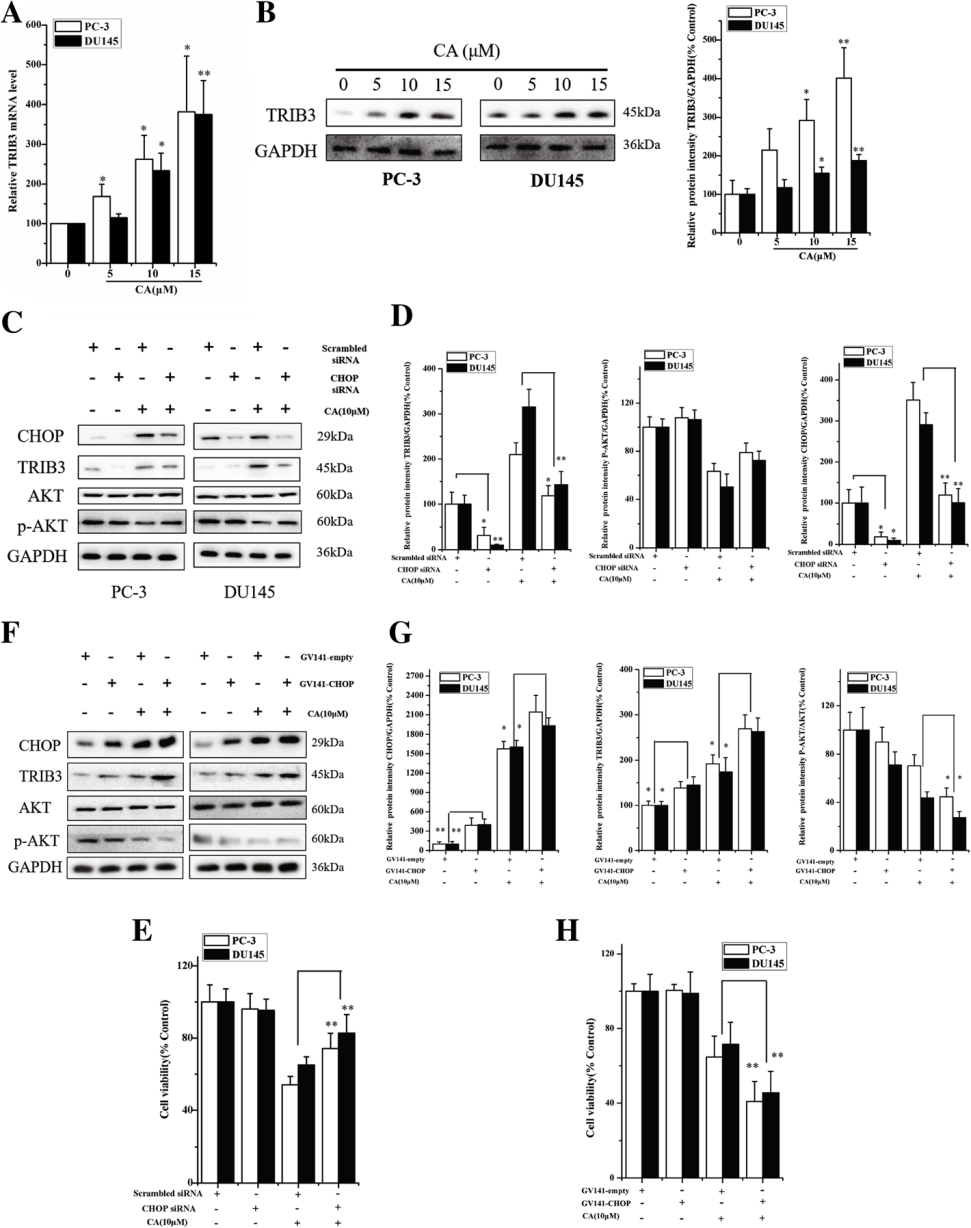
Downregulation of AKT phosphorylation seems to be a secondary event associated with the PERK/CHOP/TRIB3 pathway activation. Gene and protein levels of TRIB3 in the PC-3 and DU145 cells were detected by RT-PCR (**a**) and western blot (**b**) after treatment with (5, 10, 15 μM). The cells were transfected with CHOP siRNA or control siRNA, respectively and treated with CA for 24 h. **c** The protein expression of CHOP, TRIB3 and p-AKT was measured by western blotting. **d** Protein levels were quantified by grayscale scan and the GAPDH was used as the loading control. The results are presented as mean ± SD and represent three individual experiments. *p < 0.05 and **p < 0.01 as compared with corresponding group. **e**The cell viability was determined by MTT assay. The results are presented as mean ± SD. *p < 0.05 and **p < 0.01 as compared with corresponding group. **f** Overexpression of CHOP in PC-3 and DU145 cells that were treated with CA for 24 h. CHOP, AKT, p-AKT and TRIB3 protein expressions were determined by western blot. **g** Protein levels were quantified by grayscale scan and the GAPDH was used as the loading control. **h** MTT assay demonstrated that CHOP overexpression aggravated CA-induced inhibition of cell viability in PC-3 and DU145. The results are presented as mean ± SD and represent three individual experiments. *p < 0.05 and **p < 0.01 as compared with corresponding group

The transcription factor CHOP is a key mediator in ER stress–induced apoptosis. Excessive or aberrant ER stress results in CHOP activation to induce cell injury or death. Therefore, we assessed whether CA could activate another route of ER stress-induced apoptosis by PERK-eIF2a-ATF4-CHOP signal pathway. The results showed that Bip, p-PERK, p-eIF2α, and ATF4 protein expression, related to ER stress signaling markers, was significantly up-regulated at 24 h after CA treatment in PC-3 and DU145 cells. In addition, no significant difference was found in the total PERK and eIF2α. More importantly, an increased expression of the transcription factor CHOP was observed (Fig. 5a and b). Similar to protein expression, RT-PCR results showed that the amount of CHOP mRNAs was increased 1.33 ± 0.25, 6.38 ± 1.62, and 14.34 ± 2.52 -fold in PC-3 as well as 1.47 ± 0.36, 5.20 ± 1.83 and 8.64 ± 1.65 -fold in DU145 cells at 5, 10 and 15 μM CA, respectively (Fig. 5c). In addition, CHOP localization in human PCa cells was evaluated by immunofluorescence, revealing that CA induced a strong CHOP expression in cytoplasm and nucleus (Fig. 5a). In order to further verify that ER stress induced-CHOP plays an important role in the induction of apoptosis by CA, PERK and CHOP were silenced using siRNA. Transfection with PERK siRNA led to a significant decrease of total PERK levels and inhibited CHOP activation induced by CA, compared to CA treated cells transfected with negative siRNA (Fig. 5e and f). Moreover, the data showed that PERK siRNA significantly attenuated CA apoptotic induction on both PC3 and DU145 cells as shown by MTT (Fig. 5g). Furthermore, CHOP knockdown by siRNA rescued CA-mediated cell death, compared to that in control cells treated with CA (Fig. 6e). However, overexpression of PERK partially upregulated CHOP levels and aggravated CA-induced apoptosis of PC-3 and DU145 cells (Fig. 5h, i and j). Meanwhile, the overexpression of CHOP in PC-3 and DU145 cells promoted CA-induced apoptosis (Fig. 6f, g and h). Therefore, these results demonstrated that CA increased CHOP activation and induced apoptosis via the PERK-eIF2a-ATF4 of ER stress-dependent apoptosis signaling pathway.

### Downregulation of AKT phosphorylation seems to be a secondary event associated with the PERK/CHOP/TRIB3 pathway activation

To delineate the relationship between ER stress and AKT in CA-induced apoptosis, we examined tribbles homolog 3 (TRIB3) gene and protein expression (CHOP downstream target gene) by RT-PCR and western blot after exposure of human PCa cells to CA (5, 10 and 15 μM) for 24 h. As shown in Fig. 6a and b, CA induced a strong TRIB3 mRNA and protein expression in a concentration-dependent manner. Transfection with CHOP siRNA decreased CA-induced TRIB3 protein expression in PC-3 and DU145 cells (Fig. 6c and d). In addition, CHOP knockdown in human PCa cells blunted the inhibitory effect of CA on AKT phosphorylation. (Fig. 6c and d). Meanwhile, transfection with GV141-CHOP enhanced CA-induced TRIB3 protein expression and further suppress AKT phosphorylation (Fig. 6f and g). Thus, evidence was also indicated that TRIB3 was closely correlated with the CHOP levels and CHOP activation resulted in TRIB3 up-regulation to further suppress AKT activation. Overall, these data suggested that CA exerted anti-cancer effects via reduction of AKT by increasing CHOP/TRIB3 expression, besides CA induced ER-stress-CHOP dependent apoptosis.

### CA retards tumor growth in a murine xenograft model by activating ER stress

**Fig. 7:**
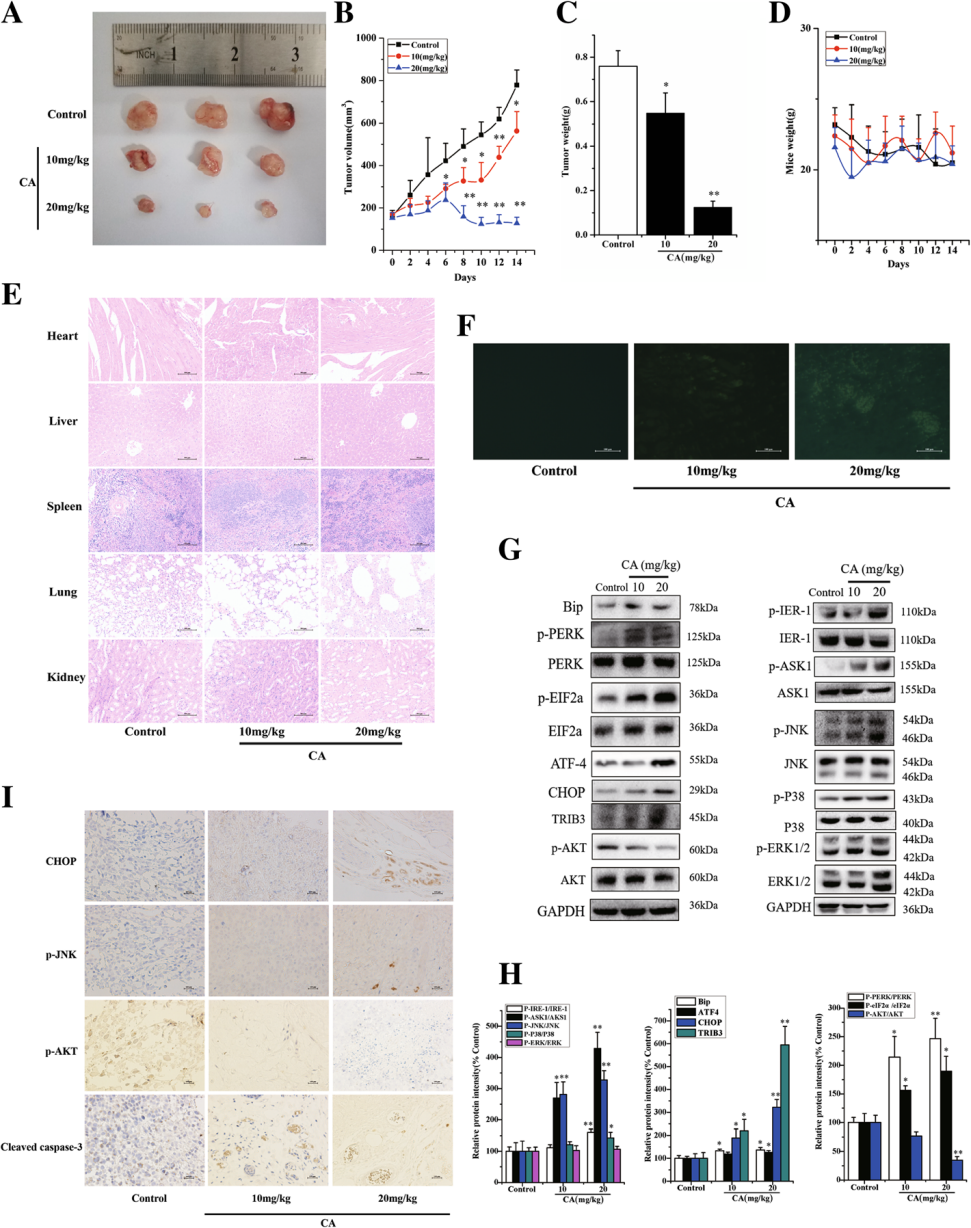
CA retards tumor growth in a murine xenograft model by activating ER stress. Typical tumor tissue images after treatment was shown in (**a**). The tumor volume (**b**), weight (**c**) and body weight (**d**) were recorded after CA treatment for 14 days. **e** Histological analysis [stained with H&E 200×] of normal tissue (heart, liver, spleen, lung and kidney) in mice induced by CA for 14 days. **f** In situ detection of apoptotic cells in tumor sections after the treatment of CA for 14 days by optical microscope using the TUNEL assay (apoptotic cell: green fluorescence). **g** The protein expression of tumor tissue was measured by western blotting. **h** Protein levels were quantified by grayscale scan and the GAPDH was used as the loading control. **i** Effect of CA on p-JNK, CHOP, p-AKT and Cleaved caspase-3 expressions in male mice tumor sections were detected by immunocytochemistry (positive cell: claybank). The results are presented as mean ± SD. *p < 0.05 and **p < 0.01 as compared with control group

To determine whether CA slows tumor progression in vivo, 2 × 10^6^ PC-3 cells were subcutaneously injected into the flanks of nude mice. We discovered that CA treatment markedly attenuated tumor volume compared with the control group (Fig. 7a and b). Tumor weight in CA-treated mice (0.550 ± 0.090 g in the 10 mg/kg group and 0.125 ± 0.028 in the 20 mg/kg group) was significantly less compared with the tumor in the vehicle control mice (0.759 ± 0.070 g, Fig. 7c). Data indicated that CA produced a significant inhibition of tumor growth without toxicity observed by mean body weight (Fig. 7d) and HE stained tissue sections of heart, liver, spleen, lung and kidney observed at submicroscopic level (Fig. 7e). An increase in TUNEL+ cells (green fluorescence) was found in tumors of the CA-treated mice compared with that of tumors from the vehicle-treated mice (Fig. 7f). Western blot revealed that CA treatment significantly increased phosphorylated PERK and phosphorylated eIF2α levels, up-regulated ATF4, CHOP protein expression, and subsequently activated CHOP downstream target TRIB3 (Fig. 7g and h). Further results showed a decrease in the expression of phosphorylated AKT in tumors, which was mediated by increased TRIB3 after CA treatment (Fig. 7g and h). Moreover, CA treatment increased AKS1 and JNK phosphorylation due to activated IRE-1 (Fig. 7g and h). PC-3 xenografts/tumors immunohistochemistry showed that CA induced an increase in ER stress-dependent CHOP expression as shown by immunohistochemistry. Moreover, significant increase of JNK phosphorylation and cleaved caspase-3 was also found in tumor tissue section by immunohistochemistry (Fig. 7i). Consistent with western blot, remarkable suppression of AKT phosphorylation was observed by immunohistochemistry in tumor tissue section after CA treatment (Fig. 7i). The above data suggested that CA exposure substantially suppressed PC-3 xenograft tumor growth in vivo through regulation of ER stress-mediated apoptosis via activation of IRE1α and PERK signaling.

**Fig. 8:**
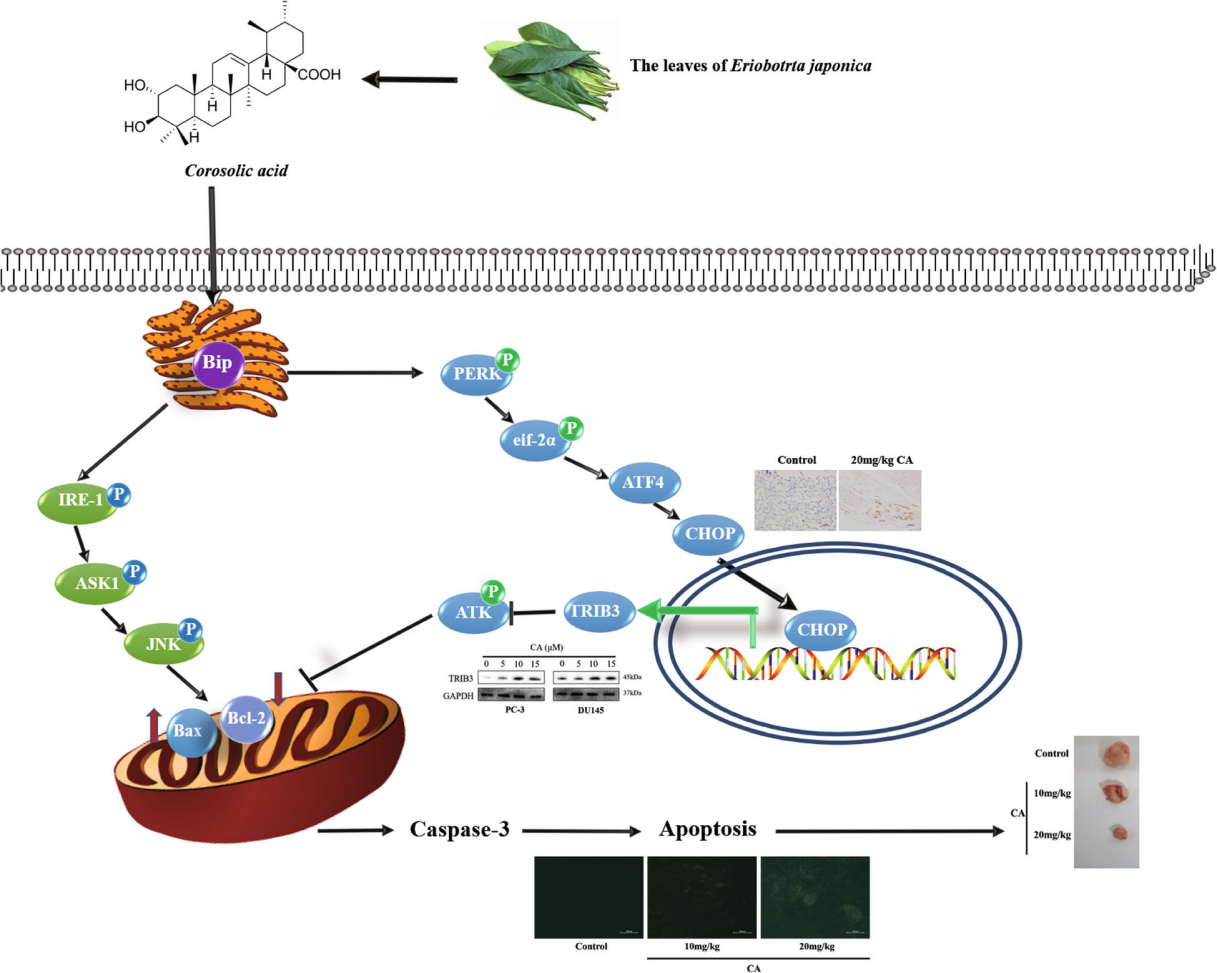
Overview of pathways for CA induced apoptosis of PCa

## Discussion

To our knowledge, the current research is the first one providing a preclinical rationale of the therapeutic effect of CA against human PCa, especially CRPC in vitro and in vivo, revealing the underlying molecular mechanisms.

In this study, we showed that CA elicited dramatic effects on the inhibition of cell proliferation and stimulation of cell apoptosis. JC-1 dye by flow cytometry revealed that mitochondrial membrane potential was indeed reduced in human PCa cells after CA treatment. In addition, our data showed that exposure of CA led to negative effects on anti-apoptotic protein Bcl2 and positive effects pro-apoptotic protein Bax levels, which are biomarkers for mitochondrial apoptotic pathway. The increase of caspase-3 was confirmed with the increased cleavage of PARP, suggesting that mitochondrion-dependent intrinsic apoptotic pathways participated in CA associated cytotoxicity. CA-induced apoptosis was not completely blocked by a caspase inhibitor when CA was used at high concentration, suggesting that a caspase-independent pathway could be also involved. Therefore, the potential role of CA in other cell death pathways needs further study.

MAPKs expression is closely associated with various signaling pathways critically involved in the regulation of cell proliferation and apoptosis [29]. CA treatment increased the induction of p-JNK in a concentration-dependent manner and a JNK specific inhibitor prevented this effect. Although p-p38 protein was also increased after CA treatment, pre-treatment with p38 inhibitor showed no effect on cell viability, indicating that p38 activation might not play a pivotal role in CA-induced cytotoxicity. According to the results showing JNK activation closely related to apoptosis, we further study JNK upstream regulation. Previous researches demonstrated that various cytotoxic drugs inducing JNK-depend cell death were related to the generation of ROS, and ROS are upstream JNK promoters. Nevertheless, CA treatments resulted in a weak green fluorescence and no significant difference with the control group was observed in our experiment (data not show). Therefore, JNK activation was not due to ROS accumulation [30]. However, Ca^2+^ enhancement after CA treatment suggested Ca^2+^ release into the cytosol from the ER, which is an important biomarker for ER stress [31] (Additional file 5: Figure S2).

Activation of JNK can in turn activate apoptotic pathways, which could be the downstream factor in response to ER stressors. We further investigated the ER stress-dependent apoptosis signaling pathway, revealing that this process plays a key role in the anti-cancer effect of CA. IRE-1 possesses kinase activity by IRE-1-TRAF2-ASK1-JNK, which induces mitochondrion-dependent intrinsic apoptotic pathway. In our study, we demonstrated that IRE-1 activation induced by CA resulted in ASK1 phosphorylation and then JNK phosphorylation, finally triggering to mitochondrion-dependent intrinsic apoptotic pathway. JNK activity inhibition using the inhibitor SP600125 could partially recover the cell viability. Transfection with IRE-1 siRNA resulted in the suppression of phosphorylated JNK and abolishing the apoptotic effect induced by CA, further supporting the role of JNK signaling in ER stress.

Besides activation of IRE-1-ASK1-JNK signaling in response to CA treatment, CHOP, a transcriptional target of ATF4, is a recognized indicator of ER stress-induced apoptosis. Its accumulation induces apoptosis directly by repressing the expression of the anti-apoptotic protein Bcl-2. Activation of CHOP was regulated by the upstream PERK signaling pathway. The PERK/eIF2a/ATF4/CHOP route has been more widely explored and it plays a crucial role in executing ER stress-induced apoptosis in many anticancer agents [32–34]. However, the role of this pathway in CA-induced apoptosis in human PCa cells remains unclear. In the present study, our results showed that CA exposure increased Bip expression, a marker protein of ER stress, and then, activated PERK, eIF2a and ATF4 cascade process. Finally, CHOP, the ER stress-induced apoptosis mediator, was induced after CA treatment. To confirm that CHOP activity occurred in response to ER stressors, PERK knockdown by siRNA was performed, showing that it suppressed CHOP induction by CA. CHOP knockdown resulted in a significantly less apoptosis, indicating that CHOP is an important mediator of CA-inducing apoptosis. CHOP-mediated apoptosis induced by ER stressors occurs by the mitochondrial apoptotic pathway [35]. Previous observations by other laboratories indicated that Bim (pro-apoptotic protein) is transcriptionally induced by CHOP and Bcl-2 (anti-apoptotic protein) and was down-regulated at a transcriptional level by CHOP [36]. These studies suggested that CHOP could induce the pro-apoptotic protein and inhibit the anti-apoptotic protein located in the outer mitochondrial membrane to induce apoptosis.

In the present study, TRIB3 up-regulated expression was observed in CA-treated cells. TRIB3, as a novel ER stress-inducible gene, is involved in cell death by induction of ER stress. Its expression could be induced by CHOP, provoking a decrease in phosphorylation of AKT [37]. It is well established that abnormally active AKT plays a crucial role in cancer generation and progression. Previous studies have shown that administration of different anticancer agents induces cancer cell death via TRIB3 upregulation and subsequent inhibition of AKT activation. M Salazar’s group has also shown that TRIB3 deletion is closely associated with a more aggressive phenotype in various tumor types by enhancing AKT activity [38]. The tumor inhibitory role of TRIB3 is connected to ER stress and AKT signaling pathway. Subsequently, this decrease in phospho- AKT caused by TRIB3 resulted in an enhanced Bax expression in the outer mitochondrial membrane to further promote apoptosis. Our data demonstrated that CA exposure increased TRIB3 level and suppress p-AKT, and CHOP knockdown could partly downregulate TRIB3 expression and abolish p-AKT inhibition in vitro (Fig. 8). Therefore, according to the background and our results, we could conclude that CHOP might be one of the critical target proteins for CA anti-cancer action. On the one hand, CHOP activation directly led to Bax translocation into mitochondria, triggering intrinsic apoptosis. On the other hand, CA might induce apoptosis by interfering with AKT activation through ERS-mediated up-regulation of CHOP/TRIB3.

## Conclusion

In conclusion, here we showed for the first time that CA, as a potent antitumor agent, exhibited anti-cancer activity on human CRPC via induction of ER stress-dependent apoptotic signaling. CA-induced cell survival inhibition was partly regulated by activation of IRE-1-ASK1-JNK and PERK-CHOP-mediated ER stress, which subsequently triggered mitochondrion-dependent intrinsic apoptosis. The present study also presented evidences that the activated CHOP regulated TRIB3 expression resulting in p-AKT inhibition, indirectly display extinction effect in cell survival and cancer progression. This research elucidating CA anti-cancer effect and its mechanism might provide a support in the development of novel therapeutic strategies against CRPC.

## Additional files

Additional file 1: **Table S1.** Purchase and dilution condition of primary antibodies and second antibodies. (DOCX 18 kb)

Additional file 2: **Table S2.** Primer sequences for PCR amplification. (DOC 31 kb)

Additional file 3: CA inhibited cell migration and invasion of human PCa cells in vitro*. (DOCX 1464 kb)*

Additional file 4: **Figure S1.** Morphology of apoptotic cells was evaluated by fluorescence microscopy following Hoechst 33258 staining at 200 × magnification. (TIF 1097 kb)

Additional file 5: **Figure S2.** Intracellular calcium concentration after CA treatment for 12 h. (TIF 615 kb)

## Abbreviations

ASK1: Apoptosis signal regulating kinase 1α
ATF4: Activating transcription factor 4
Bip: Binding immunoglobulin protein
CA: Corosolic acid
CHOP: CCAAT-enhancer-binding protein homologous protein
CRPC: castration-resistant prostate cancer
eIF2a: Eukaryotic initiation factor-2a
ER Stress: Endoplasmic reticulum stress
IRE-1: Inositolrequiring enzyme 1
JNK: c-jun N-terminal kinase
MAPK: Mitogen-activated protein kinase
PCa: Prostate cancer
PERK: Protein kinase RNA-like endoplasmic reticulum kinase
TRIB3: Tribbles homolog 3
TUNEL: Terminal dexynucleotidyl transferase(TdT)-mediated dUTP nick end labeling
