# Supplementary material for: Corosolic acid, a natural triterpenoid, induces ER stress-dependent apoptosis in human castration resistant prostate cancer cells via activation of IRE-1/JNK, PERK/CHOP and TRIB3
Source: J Exp Clin Cancer Res. 2018 Sep 3;37:210. doi: 10.1186/s13046-018-0889-x (PMC6122202; doi:10.1186/s13046-018-0889-x)
Supplement: Supplementary file 1 — Table S1. Purchase and dilution condition of primary antibodies and second antibodies. (DOCX 18 kb) [file 13046_2018_889_MOESM1_ESM.docx]

Supplement Table 1. Purchase and dilution condition of primary antibodies and second antibodies

| **Antibody** | **Corporation** | **Dilution** | | | **Storage** |
| --- | --- | --- | --- | --- | --- |
|  |  | **WB** | **IF** | **IHC** |  |
| Caspase-3 | Cell Signaling Technology (CST) | 1:1000 |  | 1:200 | -20℃ |
| PARP | CST | 1:1000 |  |  |  |
| Cytochrome c | CST | - | 1:400 |  |  |
| Bax | CST | 1:1000 |  |  |  |
| Bcl-2  p-JNK  JNK  p-P38  P38  p-AKT  AKT  p-ERK1/2  ERK1/2  p-IRE-1  IRE-1  p-ASK1  ASK1  Bip  p-eif2α  eif2α | CST  CST  CST  CST  CST  CST  CST  CST  CST  Affinity  CST  CST  CST  Proteintech Group  Affinity  Affinity | 1:1000  1:1000  1:1000  1:1000  1:1000  1:1000  1:1000  1:1000  1:1000  1:1000  1:1000  1:1000  1:1000  1:1000  1:1000  1:1000 |  | 1:200  1:200 |  |
| p-PERK | Affinity | 1:1000 |  |  |  |
| PERK | Affinity | 1:1000 |  |  |  |
| ATF4 | Affinity | 1:1000 |  |  |  |
| CHOP | Proteintech Group | 1:1000 | 1:200 | 1:100 |  |
| TRIB3 | Proteintech Group | 1:1000 |  |  |  |
| GAPDH | Affinity | 1:3000 |  |  |  |
| HRP-linked Antibody | CST | 1:1000 |  |  |  |
