# Supplementary material for: Corosolic acid, a natural triterpenoid, induces ER stress-dependent apoptosis in human castration resistant prostate cancer cells via activation of IRE-1/JNK, PERK/CHOP and TRIB3
Source: J Exp Clin Cancer Res. 2018 Sep 3;37:210. doi: 10.1186/s13046-018-0889-x (PMC6122202; doi:10.1186/s13046-018-0889-x)
Supplement: Supplementary file 2 — Table S2. Primer sequences for PCR amplification. (DOC 31 kb) [file 13046_2018_889_MOESM2_ESM.doc]

Table 2. Sequences of primers used for PCR.

| Name | Primer sequences |
| --- | --- |
| GAPDH | Forward: 5’-TTGTGACAAAGTGGACATTGTTG-3’ |
|  | Reverse: 5’-TCTCGCTCCTGGAAGATGGTGAT-3’ |
| CHOP | Forward: 5’-GCTTCTCTGGCTTGGCTGACTG-3 ’ |
|  | Reverse: 5’-TTCCTGCTTGAGCCGTTCATTCTC-3 ’ |
| TRIB3 | Forward: 5’-AGGACTCCTGCGTGCTGACTG-3 ’ |
|  | Reverse: 5’-AGGAGGCAGCGAACCAGACAG-3 ’ |
