# Supplementary material for: Corosolic acid, a natural triterpenoid, induces ER stress-dependent apoptosis in human castration resistant prostate cancer cells via activation of IRE-1/JNK, PERK/CHOP and TRIB3
Source: J Exp Clin Cancer Res. 2018 Sep 3;37:210. doi: 10.1186/s13046-018-0889-x (PMC6122202; doi:10.1186/s13046-018-0889-x)
Supplement: Supplementary file 3 — CA inhibited cell migration and invasion of human PCa cells in vitro. (DOCX 1464 kb) [file 13046_2018_889_MOESM3_ESM.docx]

**Supplement materials 1**

**CA inhibited cell migration and invasion of** **human PCa cells *in vitro***

METHODS

1. **Cell wound healing assay**

The PC-3 and DU145 cells were seeded at a high density (1×10^5^ cells/ml) in each well of a 12-well culture plate and allowed to grow to 75% confluence in a complete medium. And then, wounded by a sterile pipette tip, and washed with PBS for several times to remove cell debris and treated in the presence or absence of CA (0, 2.5 and 5μM) with 10% serum medium for 24 h. Images were captured using a Nikon Inverted Microscope (Nikon TS100, Nikon, Tokyo, Japan).

1. **Migration and invasion assay**

Cell migration and invasion assay were evaluated in the Transwell 24-well Boyden chambers (Corning Costar) with 8.0 μm pore size polycarbonate membrane. Approximately 5×10^4^ cells/ml PC-3 or DU145 cells were plated in the top chamber with free medium containing CA (0, 2.5 and 5μM). Culture medium with 10% FBS was added to the bottom chamber. After treatment for 24h at 37 °C, the medium was removed, and the filters were washed twice with PBS, fixed with 4% paraformaldehyde for 15 min and then stained with Giemsa solution (Nanjing Jiancheng Bioengineering Institute, Nanjing, Jiangsu, China). Migrated cells that passed through the filter were photographed and counted. For in vitro invasion assay, Matrigel (BD Biosciences, San Jose, CA, USA) was added to each well at 37 °C for 6 h before cells were seeded onto the membrane. After incubating for 24 h, the filters were washed, fixed, stained, photographed and counted with similar approaches of cell migration assay.

RESULTS

The effect of CA on cell migration was investigated by ‘‘wound-healing’’ assays. After 24 h, an obvious delay in the wound healing migration assay was found in exposure of CA (2.5 and 5μM) compared with the control group (Figure A). In addition, we observed that CA treatment dramatically suppressed wound closure of PC-3 and DU145 cells, compared to the vehicle treated control cells. Meanwhile, Transwell experiments without Matrigel exhibited that CA displayed a lower ability of migration compared with the vehicle treated control cells (Figure B). To investigate the potential of CA to inhibit human PCa cells invasion, we conducted invasion assays by allowing PC-3 and DU145 cells to invade Matrigel coated Transwell membranes in the presence or absence of CA. Results showed that CA suppressed the ability of PC-3 and DU145 cells to invade into a Matrigel coated Transwell membranes (Figure C).


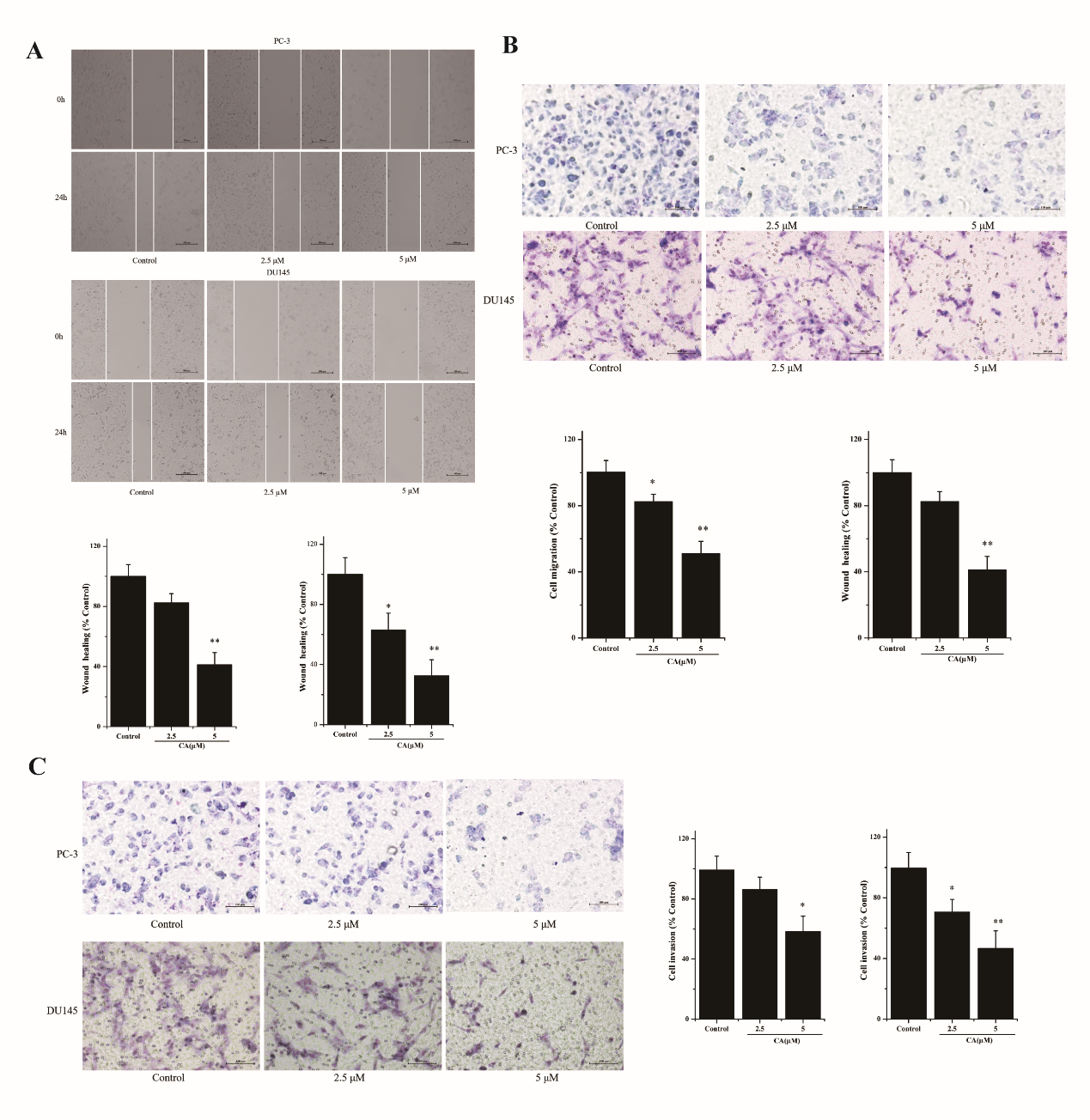


**Figure.** **CA inhibited cell** **migration and invasion of** **human PCa cells *in vitro*.**

**(A)** The inhibition of RA on PC-3 and DU145 cells were determined by wound closure assay and the column showed the quantitative results. **(B)** The migration of PC-3 and DU145 cells were investigated with transwell migration assays. Cells were treated with CA (0, 2.5 and 5μM) for 24 h and the column showed the quantitative results. **(C)** The invasion effects of CA on PC-3 and DU145 cells were measured by transwell assay with Matrigel. Cells were treated with CA (0, 2.5 and 5μM) for 24 h and the column showed the quantitative results. The results are presented as mean ± SD and described as column charts from three independent experiments were shown (*P<0.05, **P<0.01 compared with control group).
