# Supplementary figures and images for: Corosolic acid, a natural triterpenoid, induces ER stress-dependent apoptosis in human castration resistant prostate cancer cells via activation of IRE-1/JNK, PERK/CHOP and TRIB3
Source: J Exp Clin Cancer Res. 2018 Sep 3;37:210. doi: 10.1186/s13046-018-0889-x (PMC6122202; doi:10.1186/s13046-018-0889-x)

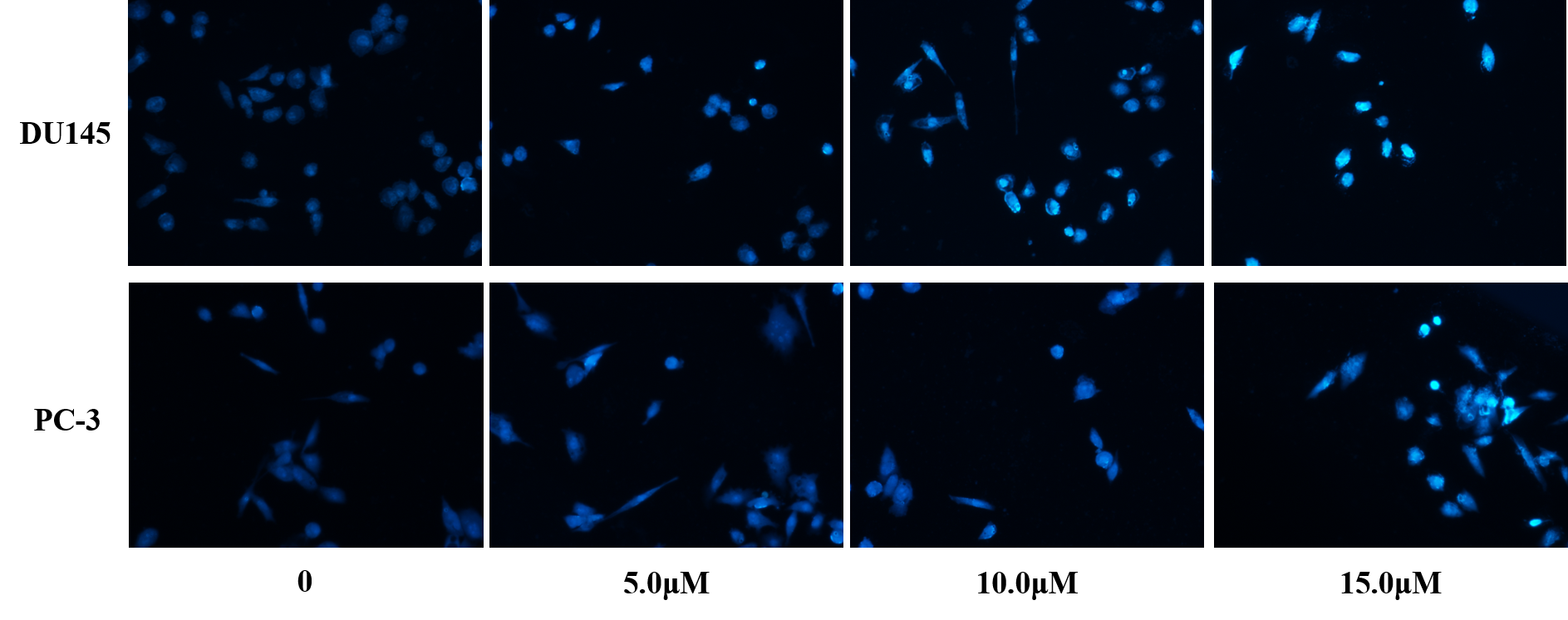

Supplement: Supplementary file 4 — Figure S1. Morphology of apoptotic cells was evaluated by fluorescence microscopy following Hoechst 33258 staining at 200 × magnification. (TIF 1097 kb) [file 13046_2018_889_MOESM4_ESM.tif]

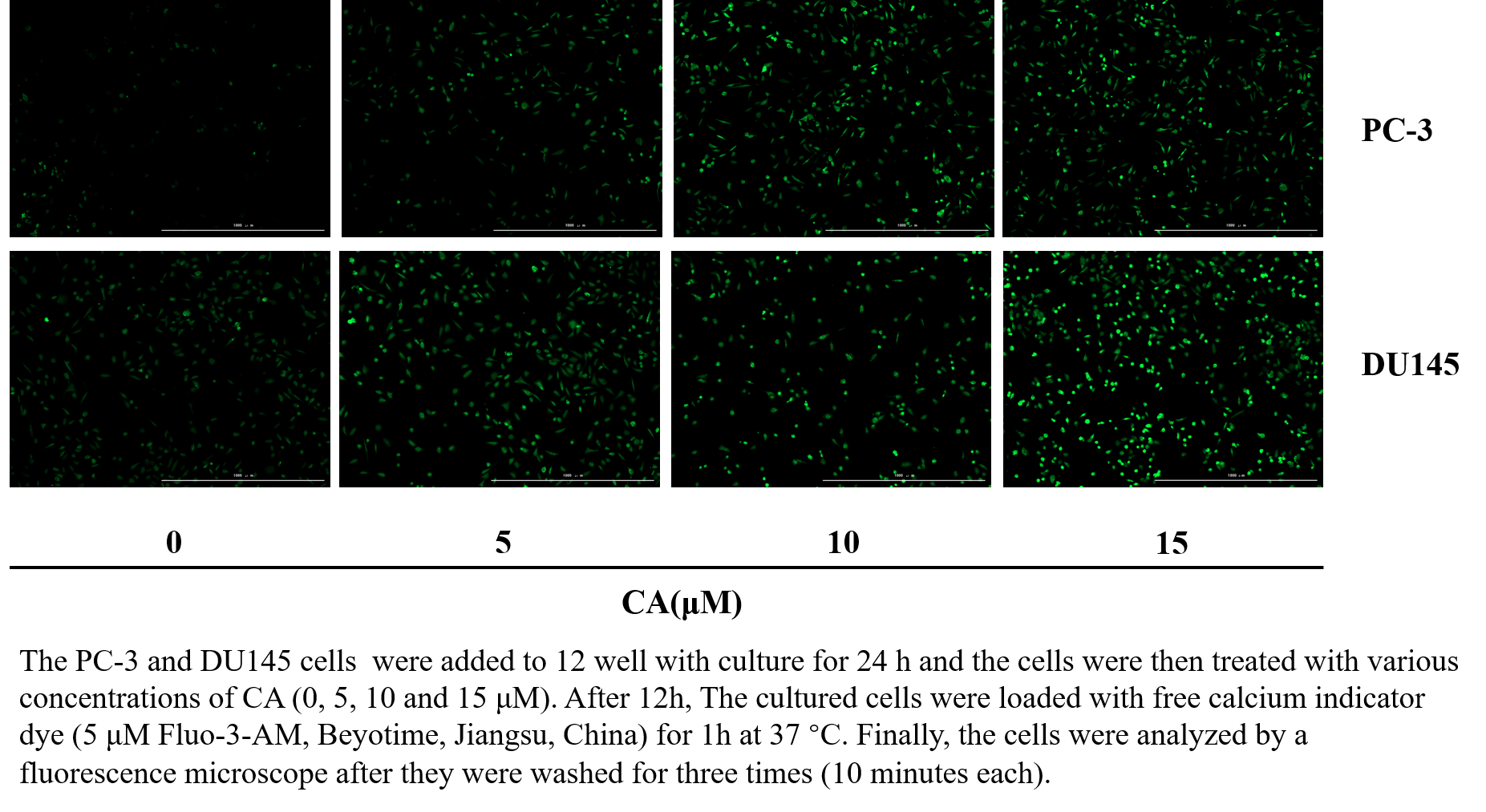

Supplement: Supplementary file 5 — Figure S2. Intracellular calcium concentration after CA treatment for 12 h. (TIF 615 kb) [file 13046_2018_889_MOESM5_ESM.tif]
